# Dickkopf-1 promotes Vascular Smooth Muscle Cell proliferation and migration through upregulating UHRF1 during Cyclic Stretch application

**DOI:** 10.7150/ijbs.56247

**Published:** 2021-03-21

**Authors:** Teng-fei Zheng, Xiao-lin Liu, Xiao Li, Qian-qian Wang, Ya-chao Zhao, Xuan Li, Meng-meng Li, Yu Zhang, Meng Zhang, Wen-cheng Zhang, Cheng Zhang, Yun Zhang, Mei Zhang

**Affiliations:** The Key Laboratory of Cardiovascular Remodeling and Function Research, Chinese Ministry of Education, Chinese National Health Commission and Chinese Academy of Medical Sciences, The State and Shandong Province Joint Key Laboratory of Translational Cardiovascular Medicine, Qilu Hospital of Shandong University,107 Wenhuaxi Road, 250012 Jinan, China.

**Keywords:** dickkopf-1, smooth muscle cell, mechanical stretch, UHRF1

## Abstract

Dickkopf-1 (DKK1) was recently shown to play an important role in cardiovascular disease. The aim of this work was to assess the role of DKK1 in the regulation of smooth muscle cell function by mechanical stretch and the mechanisms underlying this process.

**Methods:** Wild-type C57BL/6J mice were subjected to sham or abdominal aortic constriction (AAC) surgery. The expression level of DKK1 was examined by immunohistochemical staining and Western blotting. Analyses of DKK1 function in vascular smooth muscle cell (VSMC) proliferation and migration were performed. Transcriptome sequencing analysis was performed to identify the differentially expressed genes and pathways regulated by DKK1. Smooth muscle-specific Dkk1 knockout mice were used to confirm the function of DKK1 *in vivo*. Chromatin immunoprecipitation (ChIP) was used to confirm DNA-protein interactions. Promoter luciferase analysis was used to detect transcription factor activity.

**Results:** We found that AAC significantly increased DKK1 protein levels in the thoracic aorta and coronary artery *in vivo*. *In vitro*, high-level stretch (18%) induced the expression of DKK1 in VSMCs. Knocking down DKK1 inhibited VSMC proliferation and migration under high-level stretch (18%). We identified ubiquitin-like containing PHD and RING finger domains 1 (UHRF1) as a target gene of DKK1. Knockdown of UHRF1 with small interfering RNAs partially reversed the regulatory effect of recombinant DKK1 on VSMCs. Specific deletion of DKK1 in VSMCs was sufficient to attenuate the AAC-induced upregulation of UHRF1, thickening of arterial media and increase in VSMC proliferation. Furthermore, we found that DKK1 regulated UHRF1 expression through the YAP-TEAD pathway. TEAD1 and TEAD4 bound directly to the promoter of UHRF1, and blocking the YAP-TEAD interaction inhibited UHRF1 upregulation due to DKK1.

**Conclusions:** This study reveals that DKK1 mediates the mechanical stretch regulation of smooth muscle cell function by modulating UHRF1 expression through the YAP-TEAD pathway.

## Introduction

The cardiovascular system is constantly exposed to varying mechanical forces defined by blood flow and pressure. It is well documented that an increase in blood pressure (hypertension) leads to vascular remodeling 1, 2. Vascular smooth muscle cells (VSMCs) in the media of the arterial wall, which are essential for maintaining vessel homeostasis, are exposed to mechanical cyclic stretch *in vivo* 3. In response to pathologically increased stretch, VSMCs switch from a contractile to a synthetic phenotype characterized by increased proliferative and migratory activities, loss of contractility, and abnormal extracellular matrix production 1, 4. However, the molecular mechanism by which mechanical cyclic stretch modulates the function of VSMCs remains to be further elucidated.

DKK1 (dickkopf-1) is the best-studied secreted protein of the Dickkopf family. It blocks the Wnt pathway by competitively binding to low-density lipoprotein receptor-related protein (LRP)5/6 and Kremen-1 on the cell membrane 5. An increasing number of studies have revealed that DKK1 exerts tumor-promoting effects 6-8. To date, multiple DKK1-neutralizing antibodies have been developed as antitumor agents and are being evaluated in clinical trials (NCT03837353, NCT04363801, NCT02013154, NCT03395080 and NCT01457417). In recent years, DKK1 was also established as a novel mediator of cardiovascular disease. High serum DKK1 levels have been associated with ischemic stroke 9, stable angina 10, and myocardial infarction 11. Moreover, DKK1 serves as a potential biomarker for the prediction of clinical outcomes among patients with acute ischemic stroke and acute coronary syndromes 12, 13. Our previous studies demonstrated that DKK1 contributes to the development of atherosclerosis 14, 15. Targeting DKK1 therapies may not only have antitumor effects but also play protective roles in cardiovascular diseases, killing two birds with one stone. Therefore, revealing the role of DKK1 in the development of cardiovascular disease is important to guide the application of antitumor agents targeting DKK1 in tumors within the population of patients with cardiovascular disease.

We have confirmed that DKK1 expression in endothelial cells was upregulated in response to disturbed flow *in vivo* and oscillatory shear stress (OSS) treatment *in vitro*. Knockdown of DKK1 attenuated OSS-induced monocyte adhesion and endothelial impairment 15. It is unknown whether DKK1 plays a role in the regulation of smooth muscle cell function by mechanical stretch. Several other previous studies have indicated conflicting roles of DKK1 in regulating the function of human vascular smooth muscle cells 16, 17. Thus, it remains a key challenge to explore the molecular signaling pathways related to the roles of DKK1 in VSMCs.

In this study, we explored the role of DKK1 in the development of arterial remodeling under conditions of pathological stretch and the underlying mechanism *in vitro* in human aortic smooth muscle cells (HASMCs) and *in vivo* in mice. We found that DKK1 levels in mouse VSMCs were significantly increased in a pathological stretch model: mice subjected to surgical abdominal aorta coarctation (AAC). SMC-specific deletion of DKK1 in mice significantly attenuated arterial remodeling. We further found that DKK1 induced the expression of ubiquitin-like containing PHD and RING finger domains 1 (UHRF1), which plays a fundamental role in the phenotypic switching of vascular smooth muscle cells by promoting proliferation and dedifferentiation 18. Mechanistically, DKK1 promoted the expression of UHRF1 through the YAP/TEAD pathway.

## Materials and methods

### Aortic constriction

Animal experiments were approved by the Ethics Committee of Shandong University and performed in compliance with the Guide for the Care and Use of Laboratory Animals (National Institutes of Health Publication Number. 85-23, revised 1985). Mice were reared in specific pathogen-free (SPF) conditions and fed a normal chow diet. The dark/light cycle was maintained at 12:12 h. Male C57BL/6J mice (Jackson Laboratory, ME, USA) (10 weeks old, 25-30 g in weight) were anesthetized with isoflurane and placed in the supine position on warm pads. Following a midline abdominal incision, the abdominal aorta was isolated below the diaphragm and near the exit of the superior mesenteric artery. The aorta was constricted by tying a 6-0 silk suture ligature against a 28-gauge needle to yield an approximately 33.75% narrowing of the luminal diameter. The needle was then removed, and the abdomen was sutured. For the sham operation, mice were subjected to the same procedure except that the ligature was not tied.

### Heart rate and blood pressure measurement

The baseline heart rate (HR) and blood pressure (BP) were measured by the mouse-tail cuff method using an automated blood pressure analysis system (Softron BP-2010, Tokyo, Japan). The HR and BP of mice in the aorta-constriction and sham-operated groups were measured with a Millar catheter (Millar SPR-671, Texas, USA) placed in the left carotid artery.

### Generation of smooth muscle-specific Dkk1 knockout mice

Dkk1 flox/flox mice were generated by flanking exon 2 of the* Dkk1* gene with loxP sites (Figure 4). To ablate DKK1 specifically in smooth muscle (Dkk1^SMKO^), Dkk1 flox/flox mice were crossed with Tagln-Cre mice, which express Cre recombinase under the control of the Tagln promoter.

### Echocardiography

Echocardiography was performed using a Vevo 2100 Imaging System (VisualSonics Inc., Toronto, Canada). During echocardiography, the heart rate of mice was maintained between 400 bpm and 500 bpm by adjusting the depth of anesthesia with isoflurane. Using modified parasternal long-axis and short-axis, two-dimensional echocardiography was used to assess the left ventricle systolic function in M-mode. The left ventricular fractional shortening (FS, %) and ejection fraction (EF, %) were automatically calculated by the echocardiographic system. Diastolic function was evaluated by tissue Doppler echocardiography presented as the E/E' ratio. E/E' is the ratio of mitral peak velocity of early filling (E) to early diastolic mitral annular velocity (E'). Echocardiography imaging and analyses were carried out by a blinded investigator.

### Cell culture

HASMCs were obtained from ScienCell Research Laboratories (CA, USA) and cultured in smooth muscle cell medium (SMCM) at 37 °C in 5% CO2. Cells from passages 4 to 6 were used for experiments. 293T cells were obtained from the American Type Culture Collection (ATCC, VA, USA) and cultured in Dulbecco's Modified Eagle's Medium (DMEM) supplemented with 10% fetal bovine serum (FBS) and 1% penicillin/streptomycin at 37 °C in 5% CO2.

### *In vitro* mechanical stretch

Cyclic stretch was performed using an FX-5000T FlexCell Tension Plus System (FlexCell, NC, USA). Four × 10^5^ HASMCs were plated on 6-well silicon elastomer-bottomed culture plates with type I collagen coating plates (Bioflex plates), and cyclic stretch was performed using a frequency of 1.0 Hz (60 cycles/min) and an elongation of 5% or 18% for the indicated time.

### Cell proliferation *in vitro* assay

Cell proliferation *in vitro* was analyzed using an EdU kit (RIBOBIO, Guangzhou, China). Six hours before the end of cyclic stretch application, EdU labeling reagent was added to the culture medium (1:1,000). After the application of cyclic stretch, VSMCs were fixed with 4% paraformaldehyde for 25 minutes. Subsequently, a glycine solution was added to neutralize the paraformaldehyde. After increasing the permeability of the cell membrane with 0.5% Triton X-100 in PBS, the cells were incubated with Apollo staining reaction solution in the dark for 30 minutes. Cell nuclei were counterstained with Hoechst 33342. Finally, fluorescence images were taken at 200×.

Cell viability was assessed using the Cell Counting Kit-8 (DOJINDO, Kumamoto, Japan) according to the manufacturer's instructions. The absorbance at 450 nm was measured in a microplate reader (BioTek, VT, USA).

### Transwell assay

Transwell assays were performed using 24-well inserts with an 8 μm pore size (Corning, NY, USA). Briefly, 600 μl SMCM supplemented with 10% FBS was loaded into the lower Transwell compartment, whereas 2 × 10^4^ VSMCs in 200 μl FBS-free SMCM were loaded into the upper compartment after the application of cyclic stretching. After 6 h, cells penetrating to the bottom of the membrane were fixed, stained with crystal violet, and analyzed under a microscope (Ti-S, Nikon). Cells were counted from 9 random fields of view.

### Histology

Fresh tissues were fixed by incubation in 4% formalin, embedded in paraffin, and sectioned transversely at a thickness of 5 µm. Paraffin sections were deparaffinized and rehydrated. Tissue sections were stained with hematoxylin and eosin to examine the morphology of the artery. Images of sections were taken and recorded with a camera connected to a light microscope. The whole aortic wall and medial areas were analyzed with ImageJ. The medial area was defined as the area between the external and internal elastic lamina. For immunohistochemistry, antigens were retrieved by citrate at the appropriate times. Sections were rehydrated, blocked with 5% bovine serum albumin (BSA) in PBS permeabilized with 0.01% Triton X-100 in PBS, and incubated with primary antibodies (shown in Table S2) at 4 °C overnight followed by incubation with horseradish peroxidase (HRP)-conjugated secondary antibodies (Jackson ImmunoResearch Laboratories, PA, USA). Detection was subsequently performed using DAB (3, 3′-diaminobenzidine) (ZSGBBIO, Beijing, China). The sections were counterstained with hematoxylin. For fluorescence immunohistochemistry, after incubation with the primary antibody overnight, the sections were incubated with Alexa Fluor 488/594 -conjugated secondary antibodies (Abcam, Cambridge, UK). Cover slips were mounted in mounting medium (Abcam) containing the nuclear stain 4, 6-diamidino-2-phenylindole (DAPI). Staining was observed and photographed under a microscope (Ti-S, Nikon).

### SiRNA and RNA interference

Upon reaching 40-60% confluence, HASMCs or 239T cells were transfected with specific siRNA or scrambled control siRNA (GenePharma, Shanghai, China) (shown in Table S2) using Lipofectamine 2000 (Thermo Fisher Scientific, MA, USA) in Opti-MEM (Thermo Fisher Scientific). After 6 h of transfection, the medium was replaced with complete SMCM, and the cells were cultured for an additional 48 h.

### Plasmid vectors and transfection

Human YAP was cloned into the pCDNA 3.1+ vector containing the CMV promoter (Invitrogen, CA, USA). Transient transfections were performed with Lipofectamine 3000 Transfection Reagent (Invitrogen) according to the manufacturer's instructions.

### Real-Time Quantitative PCR (qPCR)

Total RNA was extracted from cells using TRIzol Reagent (Life Technologies, MA, USA), according to the manufacturer's protocol. cDNA was synthesized from 1 ng of total RNA by the PrimeScript RT Reagent Kit (TakaRa Biotechnology, Dalian, China). qPCR was performed on a CFX96 Touch™ Real-Time PCR System (Bio-Rad Laboratories, CA, USA) using QuantiNova™ SYBR® Green PCR (Qiagen, CA, USA). All experiments were repeated at least 3 independent times. Relative gene expression was determined using the 2^-ΔΔCT^ method. CT values were normalized to the internal control glyceraldehyde-3-phosphate dehydrogenase (GAPDH).

### Western blot analysis

HASMCs and tissue sample proteins were extracted using RIPA buffer containing protease inhibitors (MedChemExpress, NJ, USA). The protein concentration was measured using BCA Protein Assay Reagent (Thermo Fisher Scientific). Lysates were denatured by boiling with SDS-PAGE Buffer loading buffer (Reducing, 5×) (CWBIO, Beijing, China). The proteins and prestained protein ladder (Thermo Fisher Scientific) were resolved on 10% SDS-PAGE gels and transferred to methanol-activated polyvinylidene fluoride membranes (Millipore, MA, USA). Membranes were blocked with 5% nonfat dry milk for regular antibodies, and incubated with primary antibodies overnight at 4 °C. The next day. the membranes were incubated with HRP-conjugated secondary antibody (Jackson ImmunoResearch Laboratories) for 1 h at room temperature. Detection was performed with Immobilon ECL substrate (Millipore), and the blots were imaged with a ChemiDoc Touch Imaging System (Bio-Rad). Protein expression was quantified using Image Lab 3.0 (Bio-Rad); target protein expression was normalized to that of GAPDH or β-actin in each sample and is expressed as a percentage of the control. The primary antibodies used in the experiments are shown in Table S2.

### ELISA

DKK1 in supernatants and serum from animal experiments and HASMCs was analyzed with ELISA kits (R&D Systems, MN, USA). All kits were used according to the manufacturer's protocols.

### Transcriptome RNA sequencing

Total RNA was isolated using a RNeasy Mini Kit (Qiagen, Germany). Paired-end libraries were synthesized by using the TruSeq™ RNA Sample Preparation Kit (Illumina, USA) following the TruSeq™ RNA Sample Preparation Guide. Briefly, poly-A-containing mRNA molecules were purified using poly-T oligo-attached magnetic beads. Following purification, the mRNA was fragmented into small pieces using divalent cations at 94 °C for 8 min. The cleaved RNA fragments were copied into first strand cDNA using reverse transcriptase and random primers. This was followed by second strand cDNA synthesis using DNA Polymerase I and RNase H. These cDNA fragments then went through an end repair process, the addition of a single 'A' base, and then ligation of the adapters. The products were then purified and enriched with PCR to create the final cDNA library. Purified libraries were quantified by a Qubit® 2.0 Fluorometer (Life Technologies, USA) and validated by an Agilent 2100 Bioanalyzer (Agilent Technologies, USA) to confirm the insert size and calculate the molar concentration. Cluster was generated by cBot with the library diluted to 10 pM and then were sequenced on the Illumina NovaSeq 6000 (Illumina, USA).

### Chromatin Immunoprecipitation

HASMCs were crosslinked with 1% formaldehyde followed by the addition of 125 mM glycine. After washing, the cells were collected in centrifuge tubes, sonicated and immunoprecipitated with normal IgG, anti-Histone H3 (CST, MA, USA), anti-TEAD1 (Abcam) and anti-TEAD4 (Abcam) antibodies at 4 °C overnight. After elution and reverse crosslinking, the antibody/DNA complexes, and DNA were purified by a DNA purification kit (CST) and analyzed in duplicate by PCR using primer pairs covering a specific region of the UHRF1 promoter (Table S2).

### Luciferase reporter assay

Wild-type and mutant UHRF1-promoters were cloned downstream of the Renilla reporter gene into the PGL-3 plasmid (GenePharma) and transfected into YAP1 overexpressing 293T cells and the relevant controls, using Lipofectamine 3000 (Thermo Fisher Scientific). 3′UTR site-specific mutagenesis at the predicted sites for each target was performed using the QuikChange Site-Directed Mutagenesis Kit, as described by the manufacturer (Agilent, Beijing, China). Cells were harvested 48 h after transfection and analyzed with a Dual-Luciferase Reporter Assay Kit (GeneCopoeia, MD, USA), according to the manufacturer's protocol.

### Statistical analysis

Data are presented as the mean ± SEM of at least three independent experiments. Unpaired Student's t-test, one-way ANOVA and two-way ANOVA followed by Turkey's post hoc test were used for statistical analysis. P<0.05 was considered statistically significant. Prism 6 software (GraphPad, CA, USA) was used for statistical analysis.

## Results

### Mechanical overstretch increased DKK1 expression in VSMCs

**Figure 1:**
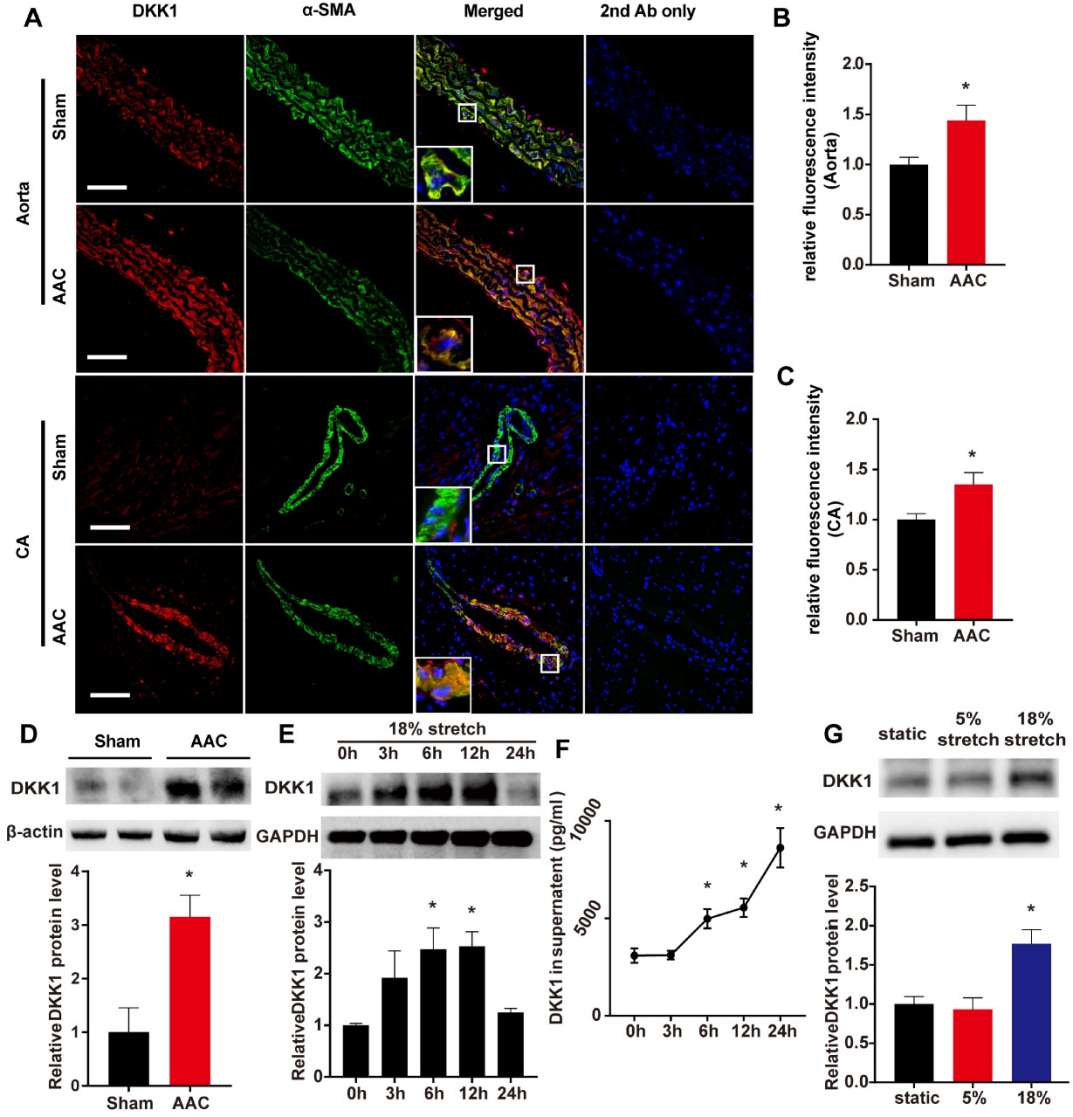
** DKK1 expression is induced after AAC in VSMCs *in vivo* and under mechanical stretch *in vitro*.** (**A**) The thoracic aorta and heart from sham or AAC mice were harvested from adult male C57BL/6 mice 7 d post-operation and analyzed by immunofluorescence (IF) staining for DKK1 (red) or SMA (smooth muscle α-actin; green). Nuclei were counterstained with DAPI (4',6-diamidino-2-phenylindole; blue). Bar=50 µm. The relative fluorescence intensities of DKK1 in smooth muscle cells of the thoracic aorta (**B**) and coronary artery (**C**) are shown. N=6, *P<0.05, vs the sham group. (**D**) DKK1 expression in the thoracic aortas from sham or AAC mice was analyzed by Western blotting. N=3, *P<0.05, vs the sham group. (**E**) Quantification of DKK1 protein expression in HASMCs treated with high-level stretch (18%) for various lengths of time. N=3, *P<0.05, vs the 0 h group. (**F**) The DKK1 protein levels in the culture supernatant of HASMCs treated with high-level stretch (18%) for various lengths of time. N=3, *P<0.05, vs the 0 h group. (**G**) Quantification of DKK1 protein expression in HASMCs under static conditions, normal stretch (5%) and high-level stretch for 6 h. N=3, *P<0.05, vs the static group.

The systolic blood pressure (SBP) and diastolic blood pressure (DBP) of AAC mice were markedly higher than those of sham-operated control mice [(SBP, 122.3 ± 4.298 vs. 87.37 ± 1.68 mm Hg; DBP, 77.14 ± 2.467 vs. 60.82 ± 1.662 mm Hg)]. As shown in Fig. 1 A-C, a significant increase in the DKK1 protein level was detected in the media of the thoracic aorta and coronary artery in the AAC mice compared with the sham-operated control mice at 1 week after the operation. Thoracic aortas were harvested and analyzed by Western blotting, and the relative protein expression levels of DKK1 were significantly increased in AAC mice (Fig. 1D). The DKK1 levels in mouse serum were determined by ELISA. No difference in serum DKK1 level was observed between the two groups (Fig. S1).

Further *in vitro* experiments were performed on human VSMCs to verify the change in DKK1 in response to high-level stretch. Using a cyclic stretch-loading system, HASMCs were treated with high-level stretch (18%) for various lengths of time (0, 3, 6, 12, or 24 h); both HASMCs and the culture supernatant exhibited time-dependent increases in DKK1 protein levels (Fig. 1E and F), while no change was observed when the cells were under 5% cyclic stretch (Fig. S2). HASMCs were treated for 6 hr with no stretch, normal stretch (5%) or high-level stretch (18%), and then cell lysates were harvested for Western blot analysis. DKK1 expression was significantly higher in cells treated with high-level stretch (P<0.05, Fig. 1G).

### DKK1 contributes to the regulation of VSMC function under mechanical stretch

**Figure 2:**
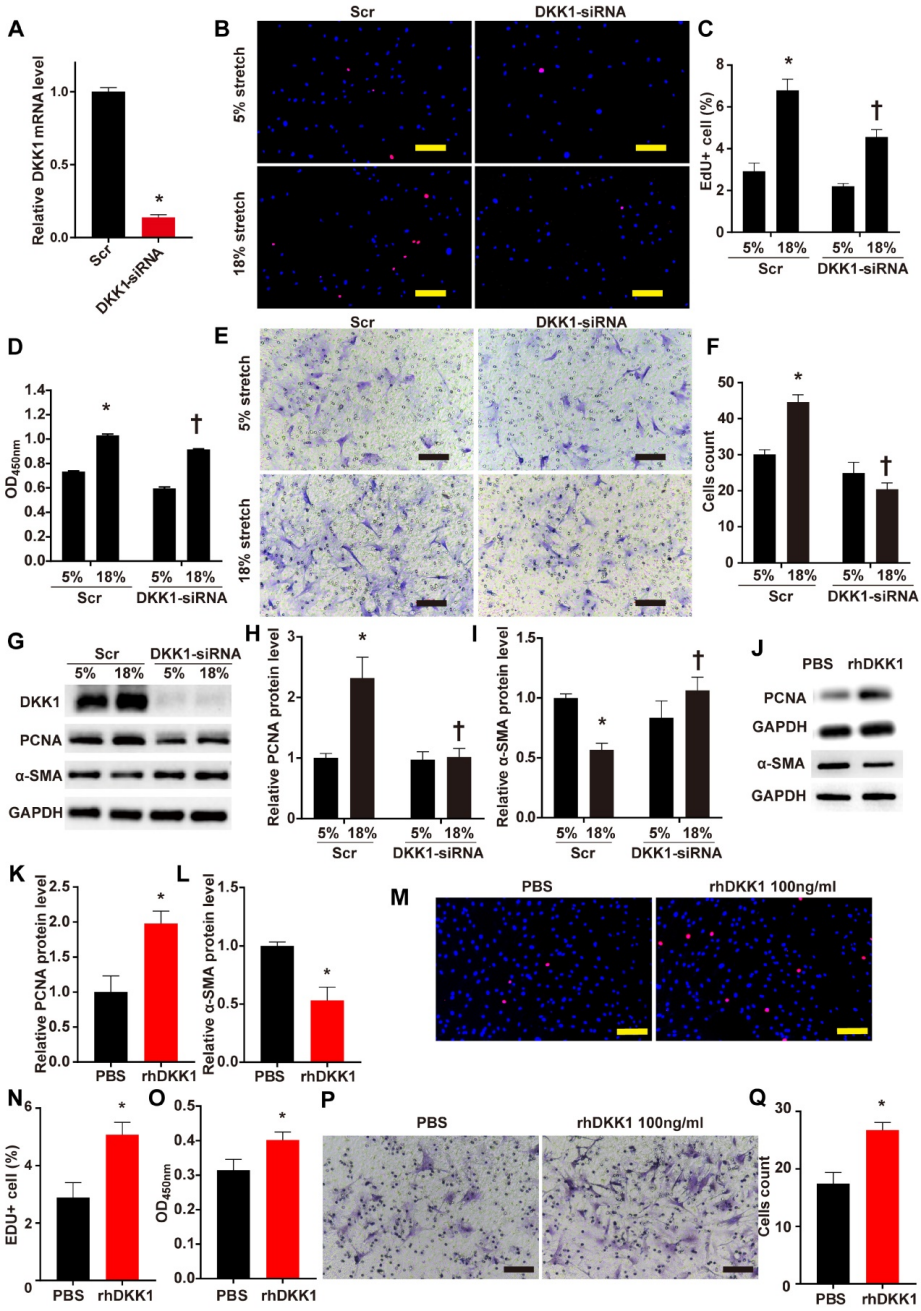
** DKK1 knockdown reduced proliferation and migration in HASMCs under mechanical stretch.** (**A**) Targeted siRNA transfection significantly decreased the expression of DKK1. N=3, *P<0.05, vs the Scr group. HASMCs were transfected with scrambled siRNA (Scr) or DKK1-siRNA for 48 hr and treated with normal (5%) or high-level stretch (18%) for 24 hr. Then, (**B-D**) EdU assays and CCK-8 proliferation assays were performed to assess HASMC proliferation. Cell nuclei were counterstained with DAPI (blue). Bar=200 µm; n= 3, *P<0.05 versus the 5%+Scr group; †P<0.05 versus the 18%+Scr group. (**E-F**) A Transwell assay was performed and the number of migrating cells was quantified. Bar=100 µm; n= 3, *P<0.05 versus the 5%+Scr group; †P<0.05 versus the 18%+Scr group. (**G-I**) Cell lysates were harvested for Western blotting analysis, relative protein expression levels of PCNA and α-SMA were plotted. (**J-L**) PBS or 100 ng/ml rhDKK1 treatment for 24 hr was performed. Then, cell lysates were harvested for Western blot analysis, and the relative protein expression levels of PCNA and α-SMA were plotted. N=3, *P<0.05 versus the PBS group. (**M-O**) PBS or 100 ng/ml rhDKK1 treatment for 24 hr was performed, and then EdU assays and CCK-8 proliferation assays were performed to assess HASMC proliferation. Cell nuclei were counterstained with DAPI (blue). N=3, *P<0.05 versus the PBS group. (**P** and** Q**) PBS or 100 ng/ml rhDKK1 treatment for 24 hr was performed. Then, a Transwell assay was performed, and the number of migrating cells was quantified. N=3, *P<0.05 versus the PBS group.

The role of DKK1 in the regulation of VSMC function is currently unclear. We knocked down the protein expression of DKK1 in VSMCs by transfecting small interfering RNA (siRNA) against DKK1 into the cells. Transfection with DKK1-siRNA for 24 hr efficiently suppressed the DKK1 mRNA level compared to transfection with scrambled siRNA (Fig. 2A). HASMCs were stimulated with 5% or 18% stretch for 24 hr after transfection with DKK1-siRNA or scrambled siRNA then subjected to EdU and CCK-8 assays to detect cell proliferation and to the Transwell assay to detect cell migration. The EdU and CCK-8 assays indicated that 18% cyclic stretch significantly promoted the proliferation of HASMCs, and knocking down DKK1-suppressed this effect (P<0.05, Fig. 2B-D). Flow cytometric analysis of cell cycle distribution indicated that cells in G0/G1 phase increased significantly, while cells in S phase decreased significantly, after DKK1 knockdown under high-level stretch (Figure S3). Similarly, the Transwell assay results consistently indicated that the number of cells that migrated into the lower chambers was significantly lower in the DKK1-siRNA group than in the control group (Fig. 2E and F). Whole-cell lysates were examined for the protein levels of the proliferation marker PCNA and the contractile phenotypic marker α-SMA by WB analysis. Compared with the normal (5%) treatment, the high-level stretch (18%) treatment upregulated the protein levels of PCNA and downregulated the protein levels of α-SMA (Fig. 2G-I). These changes were partially reversed by knocking down the expression of DKK1. Supernatants were also collected for ELISA assaying DKK1 levels (Fig. S4). The addition of exogenous rhDKK1 promoted cell proliferation and migration, facilitated the expression of PCNA and inhibited the expression of α-SMA (Fig. 2J-Q).

### DKK1 is involved in regulating HASMC function by targeting UHRF1

Considering that DKK1 is known as an antagonist of canonical Wnt signaling, we conducted experiments to determine whether the canonical Wnt pathway is involved in the roles of DKK1 in HASMCs during cyclic stretch application. FH-535 is a small molecule inhibitor of Wnt pathway 19. Pretreatment of HASMCs with FH-535 failed to reverse the downward trend in PCNA and the upward trend in α-SMA protein expression in HASMCs transfected with DKK1-siRNA (Fig. S5A-C). EdU assays, CCK-8 assays and Transwell assays also indicated that pretreatment of HASMCs with FH-535 failed to reverse the downward trend in cell proliferation and cell migration in HASMCs transfected with DKK1-siRNA (Fig. S5D-H).

**Figure 3:**
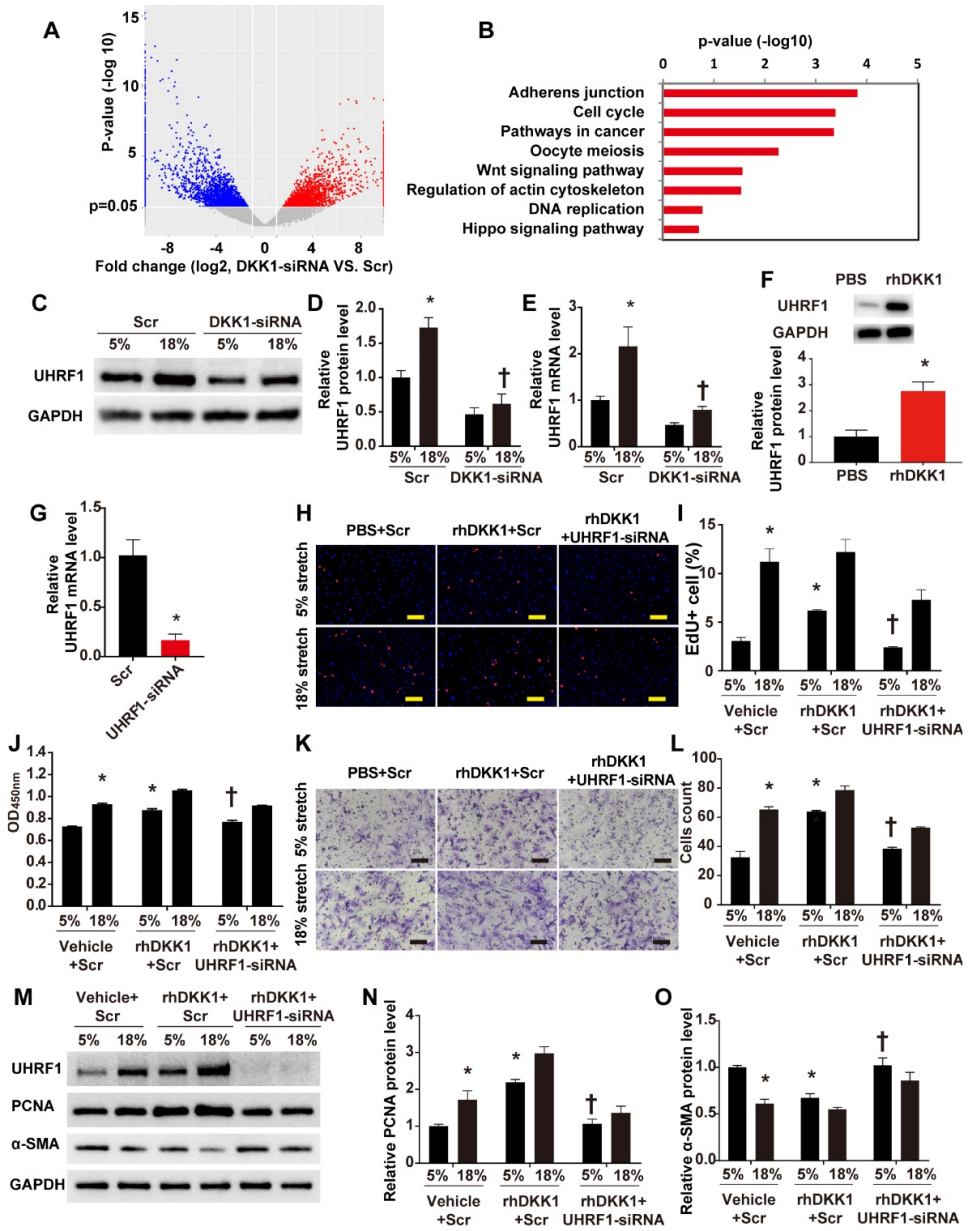
** DKK1 is involved in regulating the function of HASMCs by targeting UHRF1.** (**A**) Volcano plot of the differentially expressed mRNAs between the control and DKK1 knockdown HASMCs as revealed by RNA-sequencing and (**B**) KEGG (Kyoto Encyclopedia of Genes and Genomes) analysis. (**C-E**) HASMCs were transfected with scrambled siRNA (Scr) or DKK1-siRNA for 48 hr and treated with normal (5%) or high-level stretch (18%) for 12 hr. Then, cell lysates were harvested for Western blot analysis, and the relative protein expression levels of UHRF1 were plotted. The relative UHRF1 mRNA level was determined by RT-PCR. N= 3, *P<0.05 versus the 5%+Scr group; †P<0.05 versus the 18%+Scr group. (**F**) PBS or 100 ng/ml rhDKK1 treatment for 12 hr was performed. Then, cell lysates were harvested for Western blot analysis, and the relative protein expression level of UHRF1 was plotted. N=3, *P<0.05 versus the PBS group. (**G**) Targeted siRNA transfection significantly decreased the expression of UHRF1. N=3, *P<0.05, vs the Scr group. HASMCs were transfected with scrambled siRNA (Scr) or UHRF1-siRNA for 48 hr and treated with normal (5%) or high-level stretch (18%) in the presence or absence of rhDKK1 stimulation for 24 hr. Then, (**H-J**) EdU assays and CCK-8 proliferation assays were performed to assess HASMC proliferation. Cell nuclei were counterstained with DAPI (blue). Bar=200 µm; n=3, *P<0.05 versus the 5% +vehicle+Scr group; †P<0.05 versus the 5% +rhDKK1+Scr group. (**K-L**) A Transwell assay was performed, and the number of migrating cells was quantified. Bar=100 µm; n=3, *P<0.05 versus the 5% +vehicle+Scr group; †P<0.05 versus the 5% +rhDKK1+Scr group. (**M-O**) Cell lysates were harvested for Western blotting analysis, and the relative protein expression levels of PCNA and α-SMA were plotted.

To define the machinery involved in the roles of DKK1 on HASMCs during cyclic stretch application, we carried out transcriptome RNA sequencing. Transcriptome sequencing analyses revealed that 7,822 transcripts of 5,151 genes changed after knockdown of DKK1 under high-level stretching (Fig. 3A). KEGG pathway analysis revealed that DKK1 knockdown significantly affected the expression of genes involved in the following pathways: adherens junction, cell cycle, Wnt signaling pathway, regulation of actin cytoskeleton, DNA replication and Hippo pathway (Figure 3B). Among the differentially expressed genes, we focused on one particular gene, UHRF1. Recent findings have suggested that UHRF1 could play an important role in regulating the proliferation, migration and phenotype of vascular smooth muscle cells 18. We verified that the protein level of UHRF1 significantly increased under high-level stretch, and no change was detected under normal conditions (Fig. S6). When DKK1 was knocked down by siRNA under high-level stretch, UHRF1 expression was significantly downregulated at the mRNA and protein levels (P<0.05, Fig. 3C-E). Moreover, rhDKK1 stimulation significantly increased UHRF1 expression in HASMCs (Fig. 3F).

To further investigate whether UHRF1 mediates the effects of DKK1 on HASMCs, we knocked down UHRF1 with siRNA for 48 h and treated the cells with normal (5%) or high-level stretch (18%) in the presence of rhDKK1 stimulation for 24 h. Then we assessed cell proliferation by administering EdU and CCK-8 assays and assessed cell migration by administering the Transwell assay. The efficiency of RNA interference was evaluated by qRT-PCR (P<0.05, Fig. 3G). Knocking down UHRF1 significantly attenuated the increase in cell proliferation and migration observed in rhDKK1-stimulated HASMCs (P<0.05, Fig. H-L). Likewise, Western blot analysis demonstrated that the protein level of PCNA significantly decreased and the protein level of α-SMA increased after UHRF1 knockdown (P<0.05, Fig. 3M-O).

### Smooth muscle (SM)-specific deletion of DKK1 in mice resulted in improved vascular remodeling

**Figure 4:**
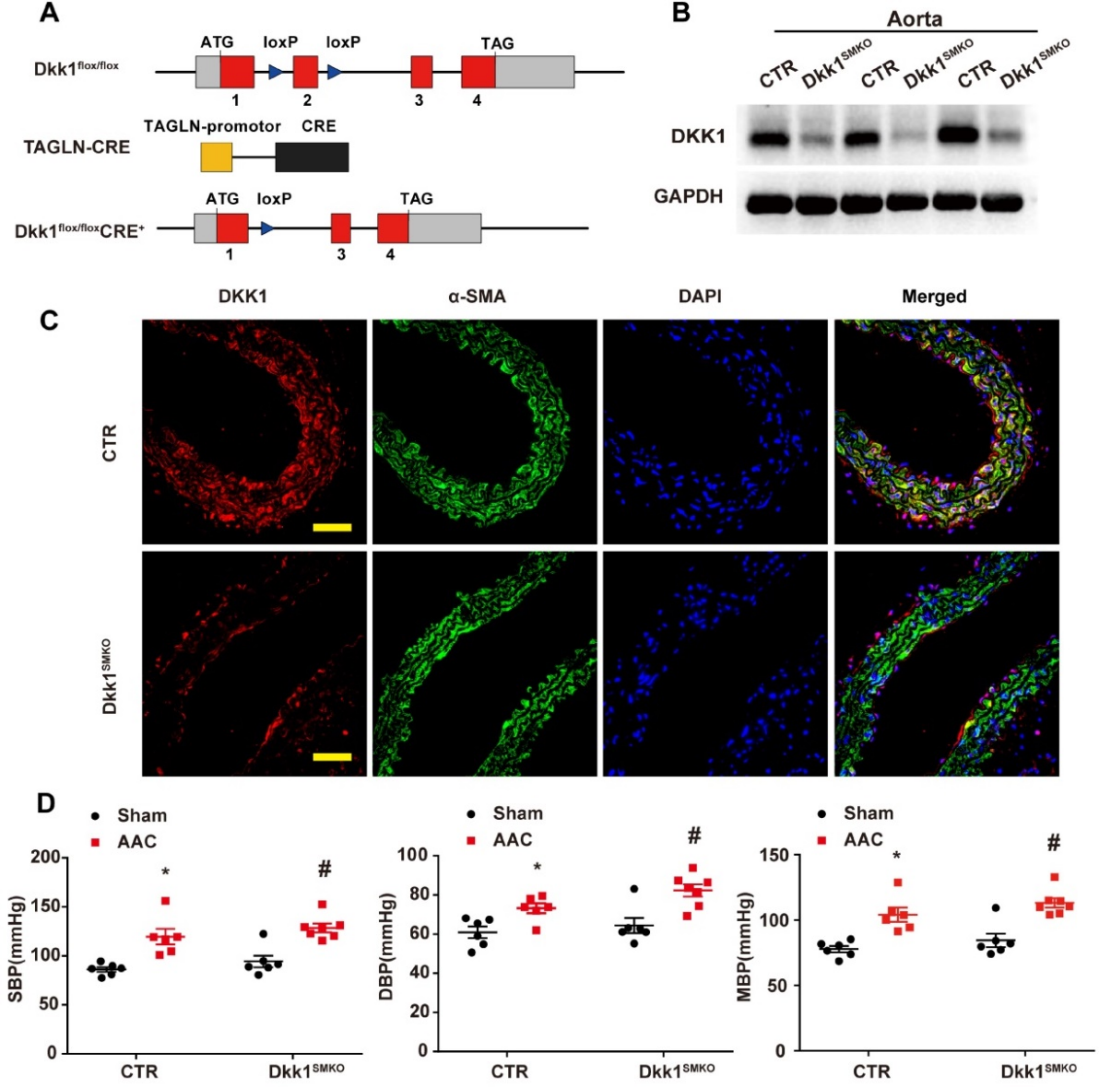
** Generation and characterization of Dkk1^SMKO^ (smooth muscle-specific Dkk1 [Dickkopf-1] knockout) mice.** (**A**) Schematic diagram of the transgenic mice used to generate Dkk1^SMKO^ mice. (**B**) Western blot analysis of the DKK1 protein levels in the aortas from the CTR and Dkk1^SMKO^ mice (n=3). (**C**) Thoracic aortas from the control or Dkk1^SMKO^ mice were isolated for immunofluorescence staining with antibodies against DKK1 (red) or SMA (SM α-actin; green). Nuclei were counterstained with DAPI (blue). Bar=50 µm. (**D**) SBP (systolic blood pressure), DBP (diastolic blood pressure) and MBP (mean blood pressure) measured with a Millar catheter placed in the left carotid artery. N= 6 or 7. *P<0.05 versus the sham+CTR group; #P<0.05 versus the sham+Dkk1^SMKO^ group.

Global homozygous deletion of DKK1 is embryonically lethal 20. To explore the role of smooth muscle DKK1 *in vivo*, Dkk1 flox/flox mice were cross-bred with TAGLN-Cre mice expressing Cre recombinase to ablate Dkk1 expression in smooth muscle (Dkk1^SMKO^; Fig. 4A-C). For all experiments, the control mice were TAGLN-CRE-negative littermates (Control). There were no significant differences between Dkk1^SMKO^ or control mice regarding body weight, blood pressure, or heart rate (Table S1).

**Figure 5:**
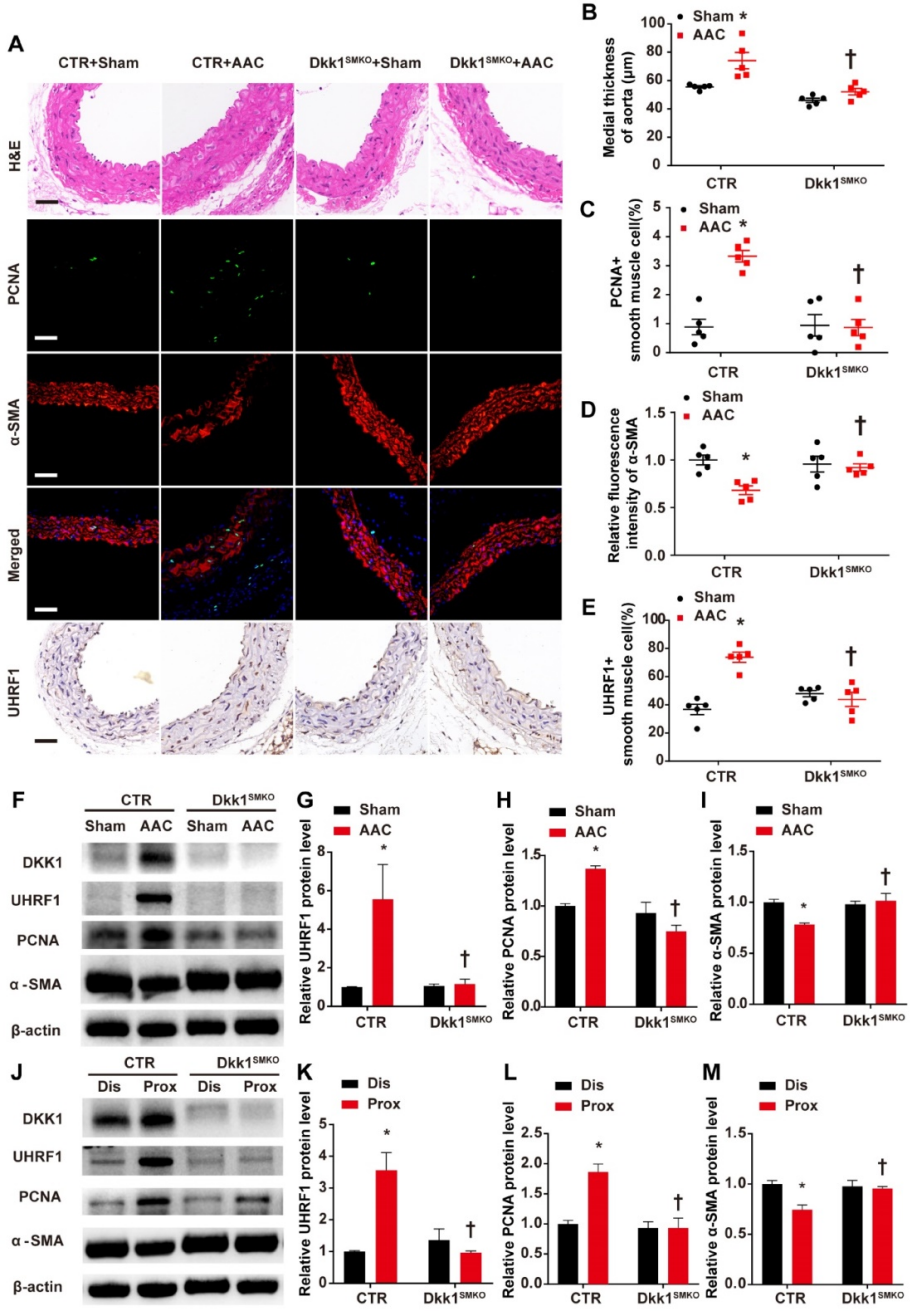
** Smooth muscle (SM)-specific deletion of DKK1 in mice resulted in improved vascular remodeling.** (**A**) Control or Dkk1^SMKO^ mice were subjected to a sham operation or AAC. Three weeks post-operation, thoracic aortas were isolated for immunofluorescence staining with antibodies against PCNA (green) or SMA (red). Nuclei were counterstained with DAPI (blue), and immunohistochemical staining was performed with antibodies for UHRF1 and HE staining. (**B**) The mean thickness of the media was determined. (**C**) The percentage of nuclei positive for PCNA in smooth muscle cells was plotted. (**D**) The relative fluorescence intensity of α-SMA was plotted. (**E**) The percentage of nuclei positive for UHRF1 in smooth muscle cells was plotted. Bar=50 µm; n=5. *P<0.05 versus the sham+CTR group; †P<0.05 versus the AAC+CTR group. (**F-I**) Control or Dkk1^SMKO^ mice were subjected to a sham operation or AAC. At 3 weeks post-operation, thoracic aortas were harvested and analyzed by Western blotting as indicated. The relative protein expression levels of UHRF1, PCNA, and α-SMA were plotted. N= 3. *P<0.05 versus the sham+CTR group; †P<0.05 versus the AAC+CTR group. (**J-M**) Control or Dkk1^SMKO^ mice were subjected to AAC. At 3 weeks post-operation, proximal and distal segments of constricted aortas were harvested and analyzed by Western blotting as indicated. Relative protein expression levels of UHRF1, PCNA, and α-SMA were plotted. N= 3. *P<0.05 versus the dis+CTR group; †P<0.05 versus the prox+CTR group.

The Dkk1^SMKO^ and control mice were randomly divided into the sham groups and the AAC groups (male, 10 weeks old; n=9 per group). There were 4 groups in total: control+sham, control+AAC, Dkk1^SMKO^ +sham and Dkk1^SMKO^ +AAC. Mice were monitored for 3 weeks after the operation and then sacrificed, and hearts and aortas were harvested. One day prior to sacrifice, blood pressure was measured with a Millar catheter placed in the left carotid artery as mentioned earlier. Blood pressure was significantly increased in the AAC mice. However, no significant difference in blood pressure was observed between Dkk1^SMKO^ mice and control mice (Fig. 4D). Serum DKK1 was detected by ELISA, and no significant difference among groups was observed (Fig. S7). HE staining showed that AAC significantly increased the medial thickness of the thoracic aorta in control mice, while Dkk1^SMKO^ markedly reduced the thickening of the media layer of aorta in the Dkk1^SMKO^ +AAC mice compared with the control+AAC mice (P< 0.05, Fig. 5 A and B). The control mice also developed marked medial hypertrophy in the coronary arteries in response to AAC. However, Dkk1^SMKO^ mice showed an attenuation of these responses to AAC (Fig. S8A and B). Next, we performed immunohistochemistry for PCNA, α-SMA and UHRF1 in the thoracic aorta and heart. The control+AAC mice had increased expression of PCNA and UHRF1 but decreased expression of α-SMA in the media layer of the aorta and coronary artery. In contrast, PCNA and UHRF1 expression was attenuated and α-SMA expression was enhanced in the media layer of the aorta and coronary artery of Dkk1^SMKO^ mice (P<0.05, Fig. 5 A and C-E, Fig. S8A and C-E). Furthermore, Western blot analysis showed that compared with sham mouse aortas, AAC mouse aortas exhibited higher PCNA and UHRF1 protein levels, and DKK1 deletion reduced the AAC-induced upregulation of PCNA and UHRF1. α-SMA showed the opposite trend among the above groups (P<0.05, Fig. 5F-I). We also compared the protein expression of PCNA, α-SMA and UHRF1 in the aorta segments proximal and distal to the aortic constriction by Western blotting. The results indicated a significant increase in the PCNA and UHRF1 protein levels and a decrease in the α-SMA protein level in the aortic segment proximal to the constriction compared with its expression in the distal segment in control mice. However, this change was significantly attenuated in Dkk1^SMKO^ mice (P<0.05, Fig. 5J-M).

We calculated the ratios of heart weight to body weight (HW/BW) and heart weight to tibia length (HW/TL) in all groups. The AAC mice had significantly higher HW/BW and HW/TL ratios than the sham mice. However, there was no significant difference in HW/BW or HW/TL between the Ctr mice and the Dkk1^SMKO^ mice. We also evaluated cardiac function using echocardiography in all groups. No difference was observed in EF%, FS%, E/E' or LVPW between the CTR mice and the Dkk1^SMKO^ mice (Fig. S9).

Collectively, the results above show that DKK1 participates in vascular remodeling and alterations in VSMC function induced by high-level stretch.

### DKK1 regulates UHRF1 through the YAP-TEAD pathway

**Figure 6:**
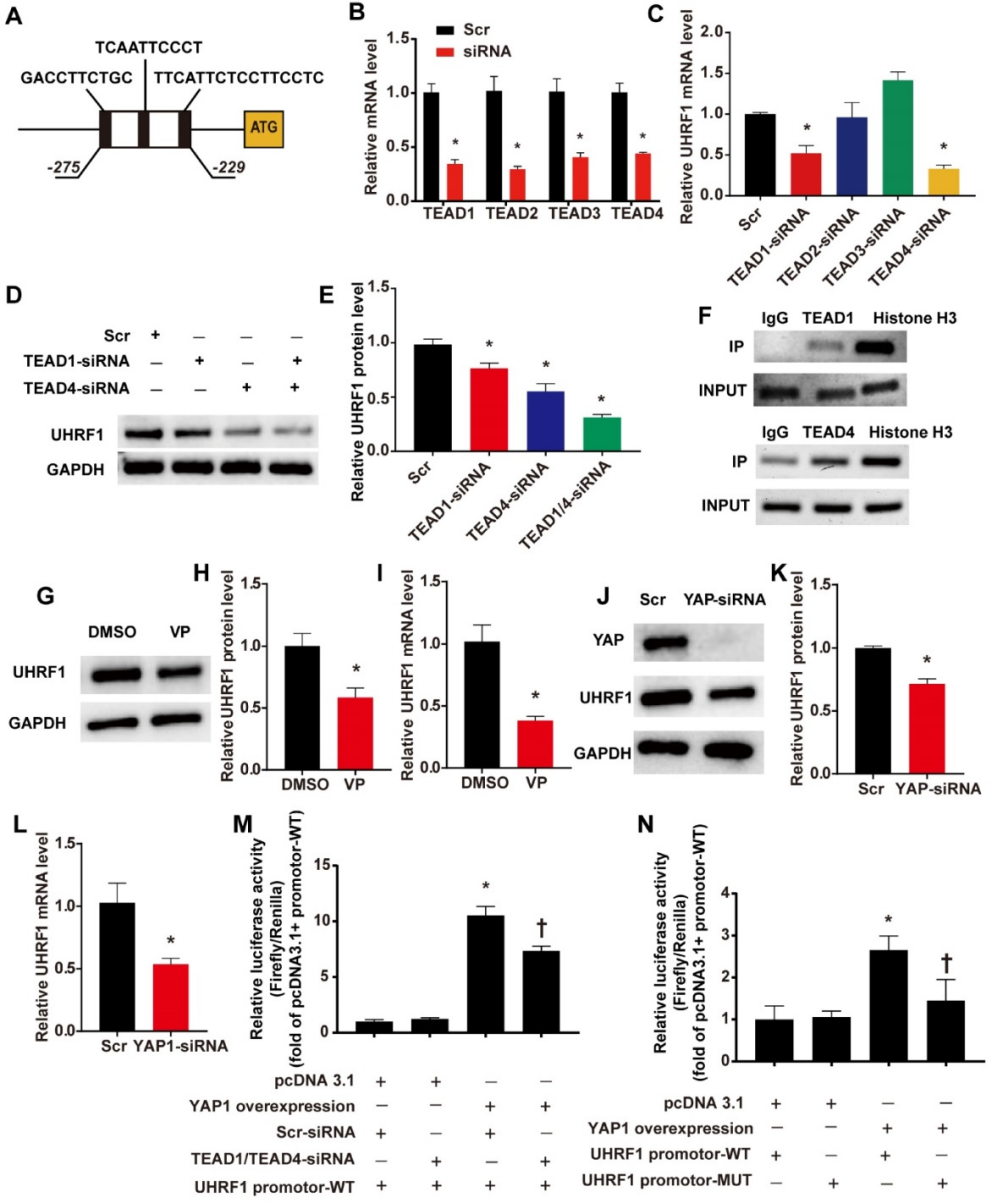
** UHRF1 is a direct target gene of the YAP-TEAD pathway.** (**A**)Three potential binding sites (named TBS1, TBS2 and TBS3) located between 275 bp and 229 bp upstream of the ATG of the *UHRF1* gene. (**B**) Target siRNA transfection significantly decreased the expression of TEAD1, TEAD2, TEAD3 and TEAD4. N=3, *P<0.05, vs the Scr group. (**C**) Under high-level stretch conditions, HASMCs were transfected with TEAD1, TEAD2, TEAD3, and TEAD4-siRNA or scrambled siRNA (Scr) for 48 hr. Total RNA was isolated for qRT-PCR analysis to determine the mRNA expression of UHRF1. N= 3, *P<0.05 versus the Scr group. (**D-E**) Under high-level stretching, HASMCs were transfected with TEAD1, TEAD4, TEAD1+TEAD4 siRNA or scrambled siRNA (Scr) for 48 hr, and then Western blotting of UHRF1 was performed. N= 3, *P<0.05 versus the Scr group. (**F**)HASMC chromatin was harvested for immunoprecipitation with an anti-TEAD1 antibody, an anti-TEAD4 antibody, an anti-histone H3 antibody or IgG control. The precipitated DNA was amplified by PCR using UHRF1 promoter-specific primers that span the potential binding region. (**G-I**) Under high-level stretch, HASMCs were treated with 10 nM verteporfin (VP) for 12 h, and then Western blotting and qRT-PCR were performed to determine the mRNA expression of UHRF1. N= 3. *P<0.05 versus the DMSO group. (**J-L**) HASMCs were transfected with scrambled siRNA (Scr) or YAP-siRNA for 48 hr and treated with high-level stretch (18%) for 12 hr. Then, Western blotting and qRT-PCR were performed to determine the mRNA expression of UHRF1. N= 3. *P<0.05 versus the Scr group. (**M**) Luciferase reporter constructs harboring the WT UHRF1 promoter were cotransfected with the YAP overexpression plasmid and scrambled siRNA or TEAD1/4 siRNA into 293T cells. Forty-eight hours post-transfection, the cells were harvested for dual luciferase assays. N=3, *P<0.05 versus the pcDNA3.1+Scr-siRNA+UHRF1 promoter-WT group; †P<0.05 versus the YAP overexpression+Scr-siRNA+UHRF1 promoter-WT group. (**N**) Luciferase reporter constructs harboring the WT UHRF1 promoter or the UHRF1 promoter with mutation of the predicted TEAD1/4-binding sites were cotransfected with the YAP overexpression plasmid or control empty plasmid into 293T cells. Forty-eight hours post-transfection, the cells were harvested for dual luciferase assays. N=3, *P<0.05 versus the pcDNA3.1+UHRF1 promoter-WT group; †P<0.05 versus the YAP overexpression+UHRF1 promoter-WT group.

We have confirmed the regulatory effect of DKK1 on UHRF1, but the mechanism behind this regulatory effect remains to be elucidated. By using the JASPAR database 21, we predicted that members of the TEAD protein family could directly bind to the UHRF1 promoter region. The results showed 3 potential binding sites (named TBS1, TBS2 and TBS3) located between 275 bp and 229 bp upstream of the ATG of the *UHRF1* gene (Fig. 6 A). Previous studies indicated that the activation of the YAP-TEAD pathway induced by biomechanical stretch can promote the phenotypic switching of SMCs 22. Therefore, we hypothesized that DKK1 regulates UHRF1 through the YAP-TEAD pathway. First, RNA interference (siRNA) was used to knock down the members of the TEAD family in HASMCs (Fig. 6B). Interference with TEAD1 and TEAD4 expression showed marked downregulation of UHRF1 mRNA (P<0.05, Fig. 6C). When we knocked down both TEAD1 and TEAD4 simultaneously, the decrease in UHRF1 protein became more pronounced than with the individual siRNA treatments (P<0.05, Fig. 6D-E). Furthermore, chromatin immunoprecipitation (ChIP) experiments demonstrated that there was an enrichment of TEAD1 and TEAD4 binding between 275 bp and 229 bp upstream of ATG of the *UHRF1* gene (Fig. 6F). When we used verteporfin (VP) to disrupt YAP-TEAD interactions or transduced HASMCs with small interfering RNA against YAP, the UHRF1 mRNA and protein levels dropped significantly (P<0.05, Fig. 6G-L). To further prove the regulatory effect of YAP-TEAD on UHRF1 promoter activity, we generated luciferase reporters harboring the UHRF1 gene promotor containing the wild-type (WT) binding region and dual luciferase assays were performed. The data from the dual-luciferase assays confirmed that YAP overexpression significantly activated WT promoter luciferase activity and that this activation was inhibited via the knockdown of TEAD1 and TEAD4 (P<0.05, Fig. 6M). To further test whether YAP-TEAD can activate UHRF1 gene promoter activity through the predicted binding region, we generated luciferase reporters harboring the UHRF1 gene promoter containing the wild type (WT) or mutant (MUT) binding region, and dual luciferase assays were performed. Data from the reporter assays showed that YAP overexpression significantly activated WT but not MUT promoter luciferase activity (P<0.05, Fig. 6N).

**Figure 7:**
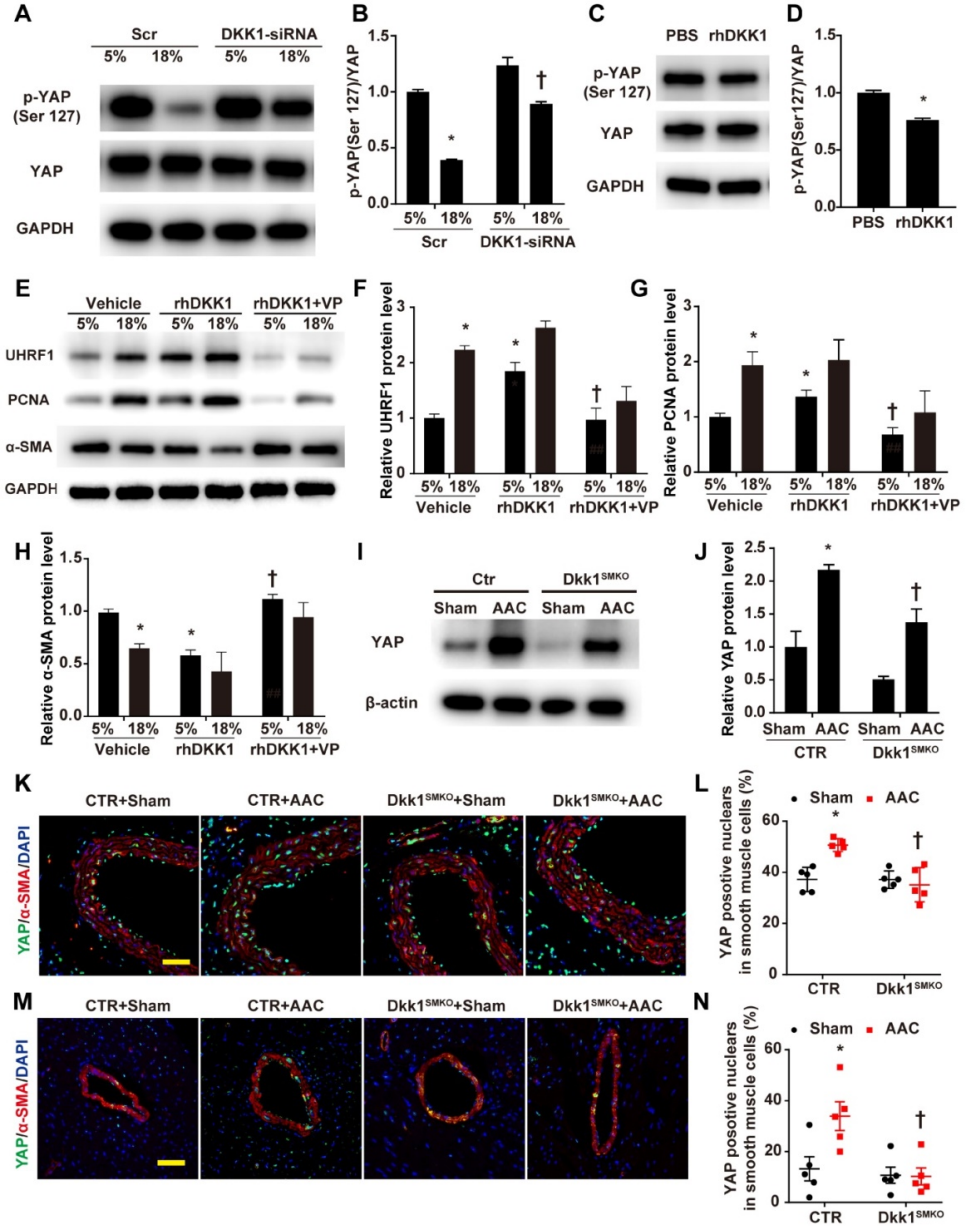
** DKK1 regulates UHRF1 through the YAP-TEAD pathway.** (**A**) HASMCs were transfected with scrambled siRNA (Scr) or DKK1-siRNA for 48 hr and treated with normal (5%) or high-level stretch (18%) for 12 hr. Then, cell lysates were harvested for Western blotting analysis, and (**B**) the p-YAP (Ser 127)/YAP ratio was plotted. N=3; *P<0.05 versus the 5%+Scr group; †P<0.05 versus the 18%+Scr group. (**C**) PBS or 100 ng/ml rhDKK1 treatment for 12 hr was performed. Then, cell lysates were harvested for Western blot analysis, and (**D**) the p-YAP (Ser 127)/YAP ratio was plotted. N=3; *P<0.05 versus the PBS group. (**E**) HASMCs were pretreated with DMSO or 10 nM verteporfin (VP) for 1 hr and then treated with normal (5%) or high-level stretch (18%) in the presence or absence of rhDKK1 stimulation for 24 hr. Then cell lysates were harvested for Western blot analysis, and the relative protein expression levels of UHRF1 (**F**), PCNA (**G**) and α-SMA (**H**) were plotted. N=3. *P<0.05 versus the 5%+Vehicle group; †P<0.05 versus the 5%+rhDKK1 group. (**I**) Control or Dkk1^SMKO^ mice were subjected to a sham operation or AAC. At 3 weeks post-operation, thoracic aortas were harvested and analyzed by Western blotting as indicated. (**J**) The relative protein expression levels of YAP were plotted. N= 3. *P<0.05 versus the Sham+CTR group; †P<0.05 versus AAC+CTR group. (**K**) The control or Dkk1^SMKO^ mice were subjected to a sham operation or AAC. Three weeks post-operation, thoracic aortas were isolated for immunofluorescence staining with antibodies against YAP (green) or SMA (SM α-actin; red). Nuclei were counterstained with DAPI (blue). (**L**) The percentage of nuclei positive for YAP in smooth muscle cells was plotted. Bar=50 µm; n= 5. *P<0.05 versus the sham+CTR group; †P<0.05 versus the AAC+CTR group. (**M**) Control or Dkk1^SMKO^ mice were subjected to AAC. Three weeks post-operation, the hearts were isolated for immunofluorescence staining with antibodies against YAP (green) or SMA (SM α-actin; red). Nuclei were counterstained with DAPI (blue). (**N**) The percentage of nuclei positive for YAP in smooth muscle cells was plotted. Bar=50 µm; n= 5. *P<0.05 versus the sham+CTR group; †P<0.05 versus the AAC+CTR group.

The above experimental results support our hypothesis that UHRF1 can be regulated by the YAP-TEAD pathway. Next, we experimentally demonstrated that DKK1 regulates UHRF1 through the YAP-TEAD pathway. Western blot analysis demonstrated that high-level stretching reduced the phosphorylation of YAP at Ser127, which was enhanced after DKK1 knockdown (Fig. 7A and B). Phosphorylation of YAP at Ser127 was also reduced in HASMCs after treatment with rhDKK1 (Fig. 7C and D). Moreover, the immunofluorescence results confirmed that DKK1 knockdown markedly reduced nuclear YAP localization (Fig. S10). VP treatment significantly reversed the effect of rhDKK1 in HASMCs, leading to a decrease in UHRF1 and PCNA expression and an increase in α-SMA expression in HASMCs (Fig. 7E-H). Western blot analysis showed that compared with sham mouse aortas, AAC mouse aortas exhibited higher YAP protein levels, and DKK1 deletion reduced the AAC-induced upregulation of YAP (P<0.05, Fig. 7I and J). Immunofluorescence detection of YAP was performed on thoracic aorta and heart sections. AAC increased the proportion of YAP-positive nuclei in vascular smooth muscle cells. In contrast, this proportion was significantly decreased in the Dkk1^SMKO^ mice (Fig. 7K-N).

## Discussion

Here, we report that DKK1 is a critical mediator of VSMC phenotypic switching induced by mechanical stretch. Our data demonstrate that high-level stretch induces the expression of DKK1 *in vivo* and *in vitro*. *In vivo*, we found that DKK1 gene deletion from SMCs alleviated vascular remodeling induced by AAC. *In vitro*, stretch-induced DKK1 upregulated UHRF1 through the YAP-TEAD pathway and resulted in enhanced VSMC proliferation and migration.

Mechanical stretch is an important cause of cardiovascular remodeling. Previous studies have shown that mechanical stretch promotes smooth muscle proliferation, migration, and phenotypic transition, which is an important mechanism of arterial remodeling in diseases that can cause pathological mechanical stretch, such as hypertension 23-25. Reports in the literature on AAC- or TAC-induced arterial remodeling are conflicting. Studies have reported a significant increase in smooth muscle cell proliferation in the thoracic aorta or carotid artery in rats at 9 days or 1 week after AAC 23, 26. An increase in the number of SMCs and the formation of neointima were observed in mini pigs at 2 weeks after AAC 27. However, other studies indicated no significant change in SMC proliferation in the aortic segment proximal to the constriction in TAC mice at 2 weeks post-operation 28, 29. In the present study, AAC mice were used for the *in vivo* study of high-level stretch, and the thoracic aorta and heart were harvested at 3 weeks post-operation. Compared with that in sham mice, the expression of proliferation markers was significantly increased in SMCs of the thoracic aorta and coronary artery and neointima formation was observed in the coronary artery in AAC mice.

Studies have shown that DKK1 plays a role in cardiovascular disease, not only by correlating with the severity of acute coronary syndromes, but also by promoting disease progression. Our group has previously found that DKK1 expression in endothelial cells is regulated by shear stress 15. This study is the first to report that DKK1 expression in SMCs is regulated by mechanical stretch. Prior studies addressing the effects of DKK1 on SMC function have yielded conflicting results. Zhu et al. reported that curculigoside A-induced SMC proliferation was counteracted by DKK1 30. Zhuang et al. reported that the proliferation-promoting effect of hyperlipidemic serum treatment on SMCs was blocked by DKK1 31. Zheng et al. reported that treatment of SIRT7-overexpressing SMCs with DKK1 increased cell proliferation and migration 32. Our study found that DKK1 promoted VSMC proliferation and migration and correlated with the loss of the contractile phenotype. We generated SMC-specific conditional DKK1 knockout mice for *in vivo* experiments. The *in vivo* results showed that deletion of DKK1 in SMCs had no effect on blood pressure but reversed the media thickening and SMC proliferation caused by AAC. This is one important finding of our study. Although AAC induced a notable elevation of blood pressure, Dkk1 knockout exerted no noticeable effect on blood pressure. We speculated that the protective effect of DKK1 deletion on vascular remodeling was BP independent.

UHRF1 is an important epigenetic regulator that plays a significant role in DNA and histone methylation. UHRF1 plays a critical role in biological processes including cell proliferation, cell cycle, and apoptosis, and is a potential target for the treatment of a variety of tumors. Recent findings have suggested that Uhrf1 can regulate the phenotype of VSMCs by promoting proliferation, migration and dedifferentiation 18. This is a new mechanism of phenotypic regulation. We found that UHRF1 expression was also significantly increased in the thoracic aorta of mice after AAC, and our *in vitro* studies showed that UHRF1 protein levels exhibited time-dependent increases with the time of high-level stretch stimulation, and that this change could be reversed by DKK1 knockout or knockdown. In addition, stimulation of SMCs with recombinant DKK1 significantly increased UHRF1 expression, suggesting that UHRF1 expression is regulated by DKK1. After knockdown of UHRF1 with small interfering RNAs, the regulatory effect of recombinant DKK1 on SMCs was partially reversed. Future studies are required to investigate the specific role of UHRF1 in overstretch-induced vascular remodeling *in vivo*.

The YAP-TEAD pathway is a mechanotransduction pathway that plays an important role in vascular remodeling and related cardiovascular diseases 33. In endothelial cells, the YAP-TEAD pathway, which regulates proinflammatory gene expression, is inhibited by unidirectional normal shear stress and activated by abnormal shear stress 34. Previous studies have indicated that biomechanical stretch (13% elongation, 0.5 Hz) can activate YAP/TAZ, which is involved in the regulation of smooth muscle cell proliferation and phenotype 22. Transcriptome sequencing results showed that DKK1 knockdown led to changes in YAP-TEAD pathways. DKK1 knockdown or knockout attenuated YAP activation due to mechanical stretch *in vitro* and *in vivo*. Recombinant DKK1 can promote YAP activation in SMCs. ChIP experiments verified the binding of TEAD1 and TEAD4 to the UHRF1 promoter region, and blockade of the YAP-TEAD interaction significantly inhibited the upregulation of UHRF1 expression caused by rhDKK1. DKK1 regulated UHRF1 expression through the YAP-TEAD pathway. To our knowledge, there have been no reports to date showing a direct association between UHRF1 expression and the YAP-TEAD pathway, and our report enriches the knowledge of the relationship between YAP-TEAD and epigenetics.

Numerous studies have confirmed that the YAP-TEAD pathway can be regulated by G protein coupled receptor (GPCR) signaling 35-37. DKK1 was confirmed to be involved in multiple GPCR signaling pathways by inducing LRP5/6 downregulation, which is independent of beta-catenin 38. DKK1 may regulate YAP-TEAD through the LRP5/6-GPCR signaling pathway. Previous studies have suggested that DKK1 can be regulated by the YAP-TEAD pathway. In MCF10A cells, wild-type YAP but not TEAD-binding deficient mutants can induce the expression of DKK1. In 3T3-L1 cells, disruption of the YAP/TAZ and TEAD interaction by VP treatment significantly reduced DKK1 mRNA, and knockdown of TEAD reduced DKK1 levels 39. In Hep-2 cells, the expression of DKK1 was significantly decreased after YAP downregulation 40. There may indeed be a feedback loop.

Deletion of DKK1 significantly reduced the overstretch load inducing smooth muscle cell dysfunction by attenuating UHRF1 expression via inhibition of the YAP-TEAD pathway. Since DKK1 is a secreted protein, our conditional knockout phenotypes may be partially masked by DKK1 supplied by adjacent tissues or residual production of DKK1 within the aortic wall. In addition, the mechanism underlying the augmentation of YAP activation by DKK1 remains to be determined. Our study suggests that targeting DKK1 is a promising therapeutic strategy for overstretch-induced vascular remodeling.

## Supplementary Material

Supplementary figures and tables.

## Abbreviations

AAC: abdominal aorta coarctation
ANOVA: analysis of variance
BP: blood pressure
BSA: bovine serum albumin
CCK-8: Cell Counting Kit-8
ChIP: Chromatin Immunoprecipitation
CMV: Cytomegalovirus
DAPI: 4',6-diamidino-2-phenylindole
DBP: diastolic blood pressure
DKK1: dickkopf-1
Dkk1SMKO: smooth muscle-specific Dkk1 knockout
DMEM: Dulbecco's Modified Eagle's Medium
ECL: Enhanced chemiluminescence
EdU: 5-Ethynyl-2'-deoxyuridine
ELISA: enzyme linked immunosorbent assay
FBS: fetal bovine serum
GAPDH: glyceraldehyde-3-phosphate dehydrogenase
HASMC: human aortic smooth muscle cell
HR: heart rate
IgG: immunoglobulin G
KEGG: Kyoto Encyclopedia of Genes and Genomes
LRP: lipoprotein receptor-related protein
MBP: mean blood pressure
MUT: mutant
OSS: oscillatory shear stress
PCNA: Proliferating Cell Nuclear Antigen
PCR: Polymerase chain reaction
SBP: systolic blood pressure
siRNA: Small interfering RNA
SIRT7: Sirtuin 7
SMCM: smooth muscle cell medium
TAC: transverse aortic constriction
TBS: TEAD binding site
TEAD: transcriptional factor TEA domain
UHRF1: ubiquitin-like containing PHD and RING finger domains 1
VP: verteporfin
VSMC: vascular smooth muscle cell
WB: Western blotting
WT: wild type
YAP: yes-associated protein
α-SMA: Alpha-smooth muscle actin
