# Supplementary material for: Dickkopf-1 promotes Vascular Smooth Muscle Cell proliferation and migration through upregulating UHRF1 during Cyclic Stretch application
Source: Int J Biol Sci. 2021 Mar 21;17(5):1234–49. doi: 10.7150/ijbs.56247 (PMC8040467; doi:10.7150/ijbs.56247)
Supplement: Supplementary file 1 — Supplementary figures and tables. [file ijbsv17p1234s1.pdf]

DATA SUPPLEMENT

Supplemental Tables

Table S1 Baseline characteristics of CTR and $DKK1^{SMKO}$ mice.

	CTR	$DKK1^{SMKO}$
Body Weight (g)	24.82 ± 0.4701. n=12	24.52 ± 0.553. n=12
Heart Rate (beat/min)	557.1 ± 21.71, n=7	552.3 ± 22.02, n=6
Systolic blood pressure (mmHg)	106.7 ± 1.262, n=7	103 ± 2.888, n=6
Diastolic blood pressure (mmHg)	67.76 ± 1.276, n=7	72 ± 1.951, n=6
Mean blood pressure (mmHg)	80.75 ± 1.052, n=7	82.33 ± 1.679, n=6

Data was expressed as means ± SEM.

Table S2 Materials and Reagents

**Materials and Reagents**	
**β-actin**	Cell Signaling Technology, 3700, WB (diluted1:1000)
**DKK1**	SantaCruz, sc-374574, WB (diluted 1:50); Abcam, ab61275, IHC (diluted1:100)
**α-SMA**	Abcam, ab5694, WB (diluted 1:1000); IHC (diluted 1:200)
**GAPDH**	Abcam, ab181602, WB (diluted 1:10000)
**PCNA**	CST, 13110, WB (diluted 1:1000); IHC (diluted 1:400)
**UHRF1**	SantaCruz, sc-373750, WB (diluted 1:100); Affinity, DF6929, IHC (diluted 1:100000)
**YAP**	CST, 14074, WB (diluted 1:2000); IHC (diluted 1:200); IF (diluted 1:200)
**Active YAP**	Abcam, ab205270, WB (diluted 1:1000)
**p-YAP (Ser 127)**	CST, 13008, WB (1:1000)
**TEAD1**	Abcam, ab133533, ChIP (diluted 1:20)
**TEAD4**	Abcam, ab58310, ChIP (diluted 1:20)
**Recombinant human DKK1 (rhDKK1)**	R&D Systems (Minneapolis, MN, USA), 100ng/ml
**Verteporfin**	MCE, CL 318952, 10 nM
**FH535**	MCE, HY-15721, 30 μM
**HASMC**	ScienCell, Cat.No. 6110
**SMCM**	ScienCell, Cat.No. 1101
**siRNA sequence**	
**Negative control (5'-3')**	UUCUCCGAACGUGUCACGUTT
**DKK1 (5'-3')**	GCUUCACACUUGUCAGAGATT
**UHRF1 (5'-3')**	GGACGAAGTCTTCAAGATT
**TEAD1 (5'-3')**	CCACTGCCATTCATAACAA
**TEAD2 (5'-3')**	GCCAGAUGCAGUUGAUUCUTT
**TEAD3 (5'-3')**	CAGCCACAUACAGGUUCUATT
**TEAD4 (5'-3')**	GGAACAAACUGUGCCUGAATT
**YAP (5'-3')**	GACAUCUUCUGGUCAGAGA
**Primers**	**Sequence (5′-3′)**
**GAPDH-RT-F**	Forward: GCACCGTCAAGGCTGAGAAC
**GAPDH-RT-R**	Reverse: TGGTGAAGACGCCAGTGGA
**DKK1-RT -F**	Forward: ATAGCACCTTGGATGGGTATTCC

**DKK1-RT -R**	Reverse: CTGATGACCGGAGACAAACAG
**UHRF1-RT -F**	Forward: GCCACGCACGCGGTTT
**UHRF1-RT -R**	Reverse: TGGCCGTCCTCCATCTGT
**TEAD1-RT -F**	Forward: CCTGGCTATCTATCCACCATGTG
**TEAD1-RT -R**	Reverse: TTCTGGTCCTCGTCTTGCCTGT
**TEAD2-RT -F**	Forward: CCGCTACATCAAGCTGAGAACG
**TEAD2-RT -R**	Reverse: GGTTGCCATTGTCTGGAAAGCC
**TEAD3-RT -F**	Forward: AGGCAGTAGATGTGCGCCAGAT
**TEAD3-RT -R**	Reverse: TCCTGGATGGTGCTGTTGAGGT
**TEAD4-RT -F**	Forward: GAAGGTCTGCTCTTTCGGCAAG
**TEAD4-RT -R**	Reverse: GAGGTGCTTGAGCTTGTGGATG
**UHRF1-ChIP -F**	Forward: CTTTCTCCTCTGGTCGTGGG
**UHRF1-ChIP -R**	Reverse: GGCATTAACGACTAGCCAAGTG

Supplemental Figures

Figure S1

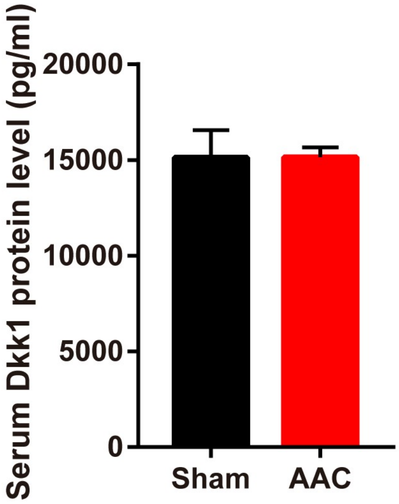

Figure S1. Dkk1 protein level in the serum from sham or AAC mice was analyzed by ELISA (n=5).

Figure S2

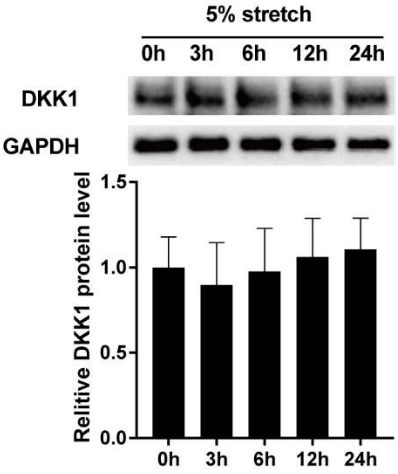

Figure S2. Quantification of DKK1 protein expression in the HASMCs treated with normal stretch (5%) for various lengths of time (n=3).

Figure S3

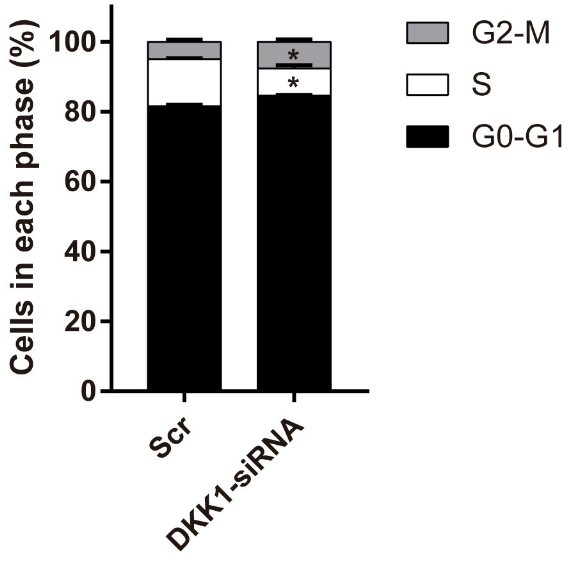

Figure S3. Flow cytometric analysis of cell cycle distribution (n=3).

Figure S4

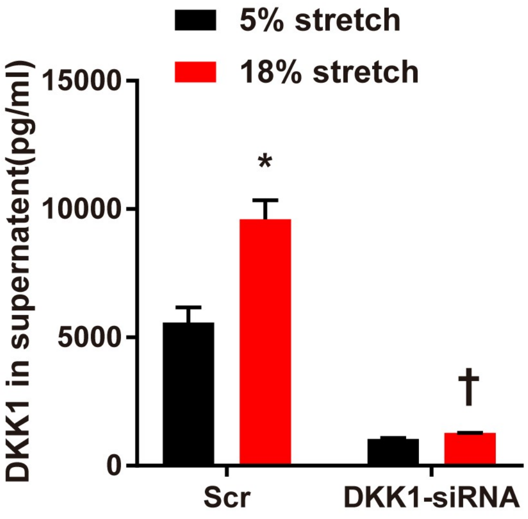

Figue S4. HASMCs were transfected with scrambled siRNA (Scr) or DKK1-siRNA for 48 h and treated with normal (5%) or high-level stretch (18%) for 24 h. Then, supernatants were collected for ELISA assaying DKK1 levels. N= 3, *P<0.05 versus the 5%+Scr group; †P<0.05 versus the 18%+Scr group.

Figure S5

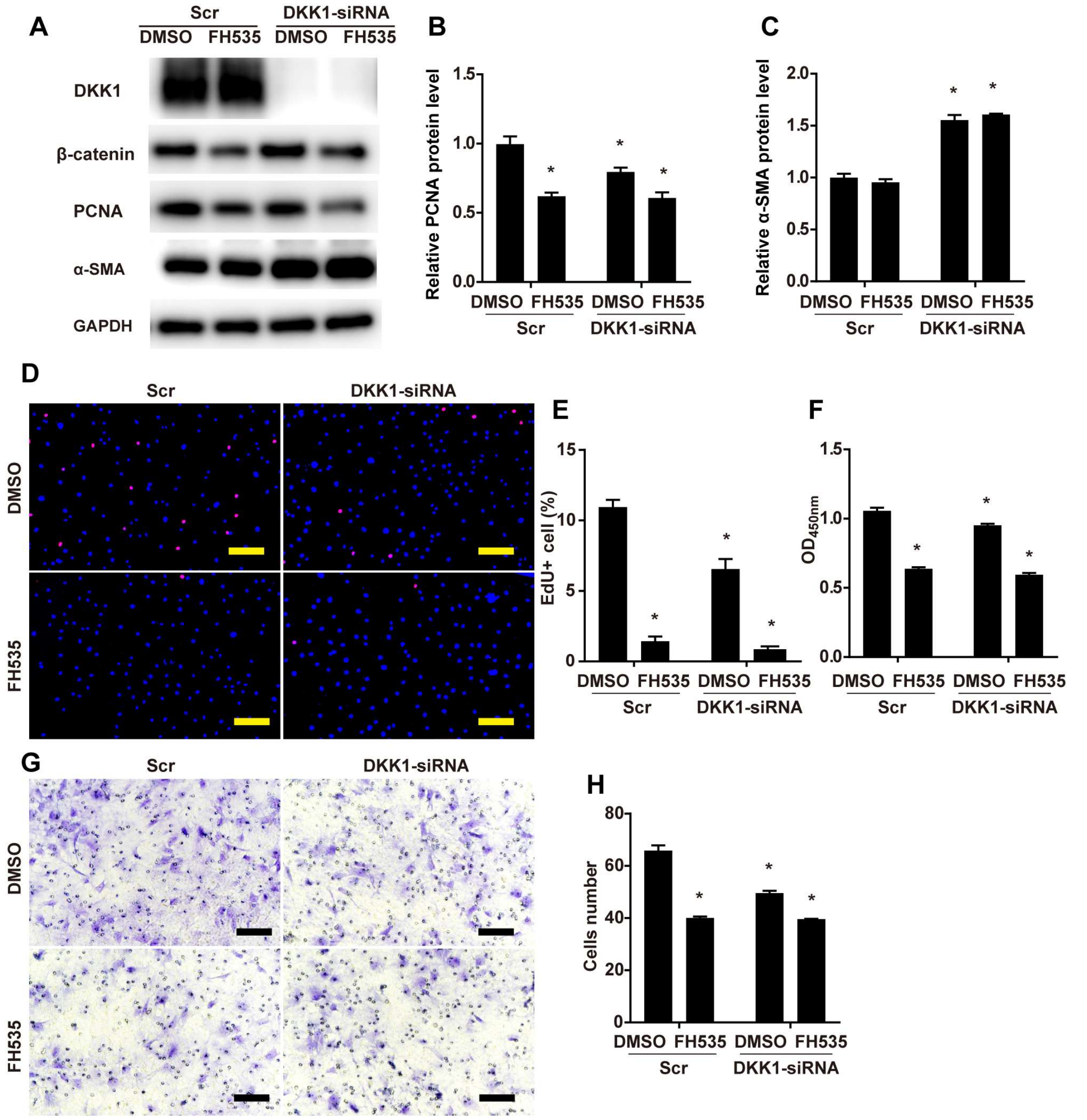

Figure S5. HASMCs were pretreated with DMSO or 30 μM FH535 for 1 hour before transfection with scrambled siRNA or DKK1 siRNA for 48 hours. Then, the cells were treated with high-level stretch for 24 hours. (A) Cell lysates were harvested for Western blot analysis, and the relative protein expression levels of PCNA (B) and α-SMA (C) were plotted. n= 3, *P<0.05 versus the DMSO+Scr group. (D-F) Cell proliferation was measured by EdU and CCK-8 assays. Cell nuclei were counterstained with DAPI (blue). Bar=200 μm; n= 3, *P<0.05 versus the DMSO+Scr group. (G-H) Cell migration was examined using Transwell cell culture chambers. Bar=100 μm; n= 3, *P<0.05 versus the DMSO+Scr group.

Figure S6

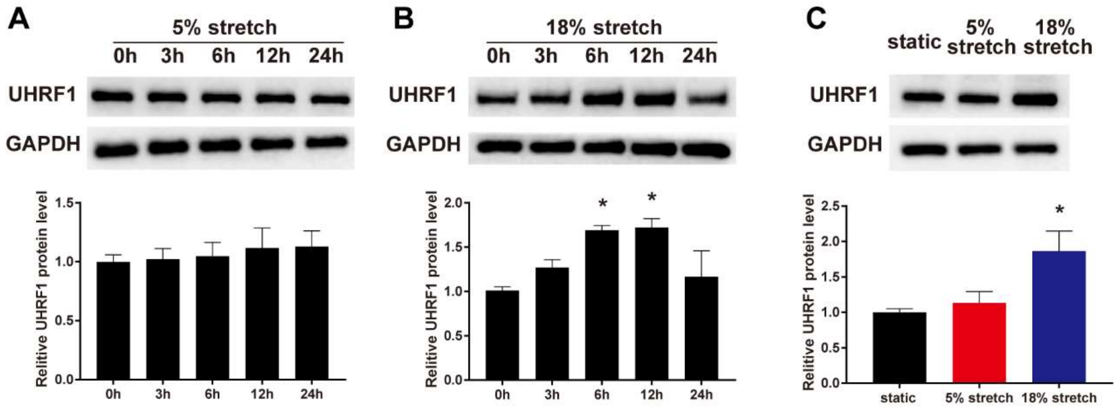

Figure S6. **Mechanical stretching can regulate UHRF1 expression in vitro.** (**A**) Quantification of UHRF1 protein expression in the HASMCs treated with normal stretch (5%) for various lengths of time (n=3). (**B**) Quantification of UHRF1 protein expression in the HASMCs treated with high level stretch (18%) for various lengths of time. N=3, *P<0.05, vs the 0h group. (**C**) Quantification of DKK1 protein expression in the HASMCs under static conditions, normal stretch (5%) and high-level stretch for 12 h. N=3, *P<0.05, vs the static group.

Figure S7

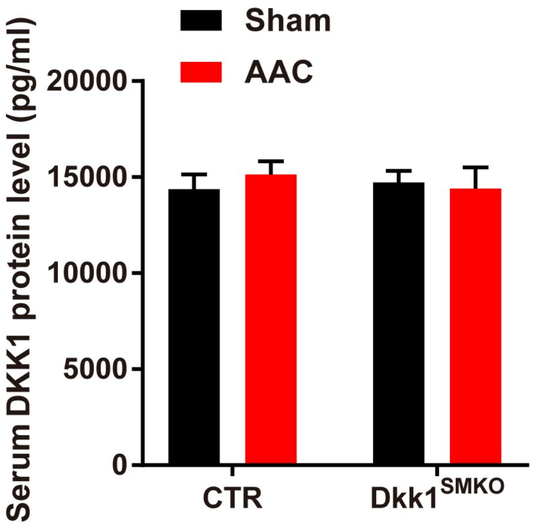

Figure S7. The control or Dkk1^{SMKO} mice were subjected to a sham operation or AAC. At 3 weeks post-operation, serum DKK1 was detected by ELISA (n=5).

Figure S8

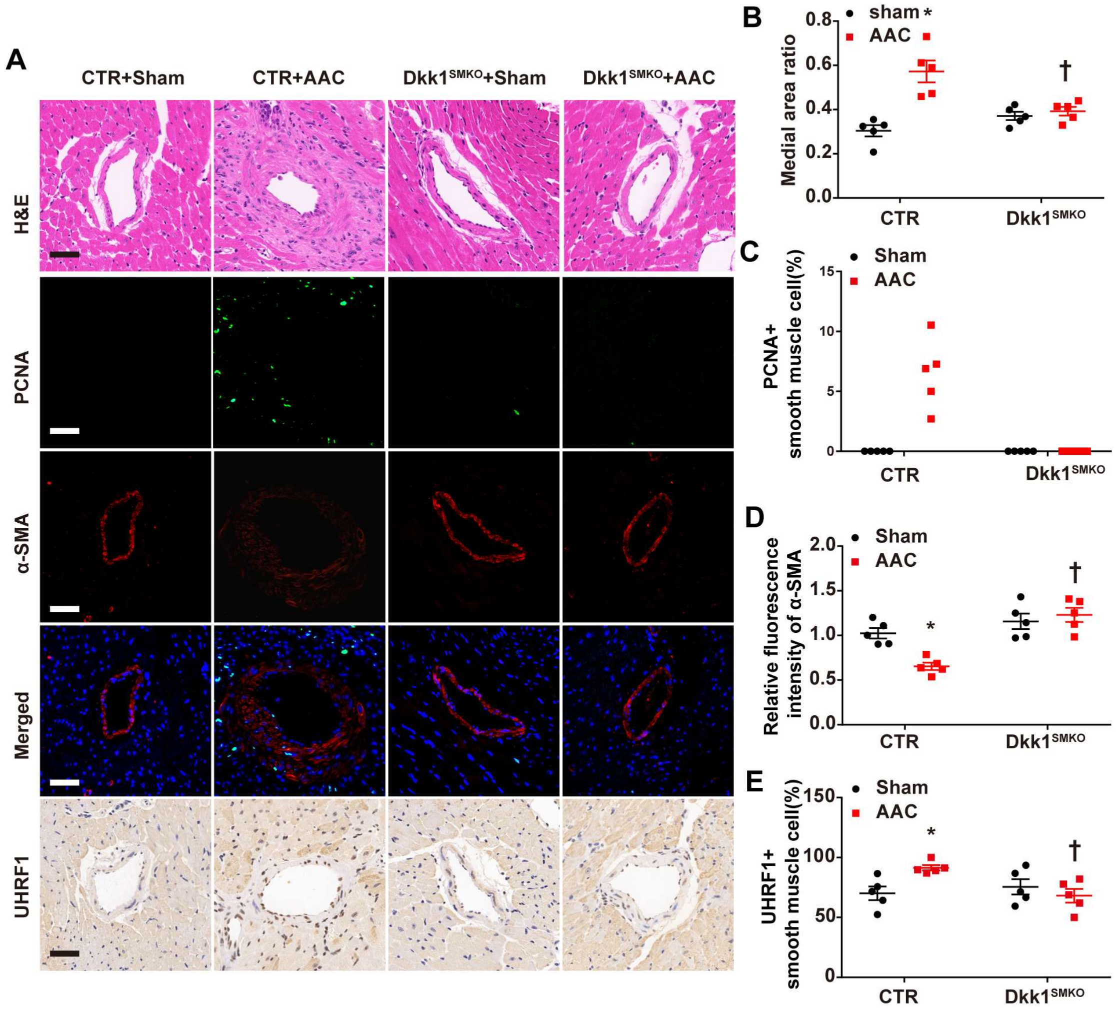

Figure S8. Smooth muscle (SM)–specific deletion of DKK1 in mice resulted in improved vascular remodeling. (**A**) The control or DKK1^{SMKO} mice were subjected to a sham operation or AAC. Three weeks post-operation, hearts were isolated for immunofluorescence staining with antibodies against PCNA (green) or SMA (SM α-actin; red). Nuclei were counterstained with DAPI (blue), and immunohistochemical staining was performed with antibodies for UHRF1 and HE staining. (**B**) The medial area ratio was determined. (**C**) The percentage of nuclei positive for PCNA in smooth muscle cells was plotted. (**D**) The relative fluorescence intensity of α-SMA was plotted. (**E**) The percentage of nuclei positive for UHRF1 in smooth muscle cells was plotted. Bar=50 μm; n=5. *P<0.05 versus the sham+CTR group; †P<0.05 versus the AAC+CTR group.

Figure S9

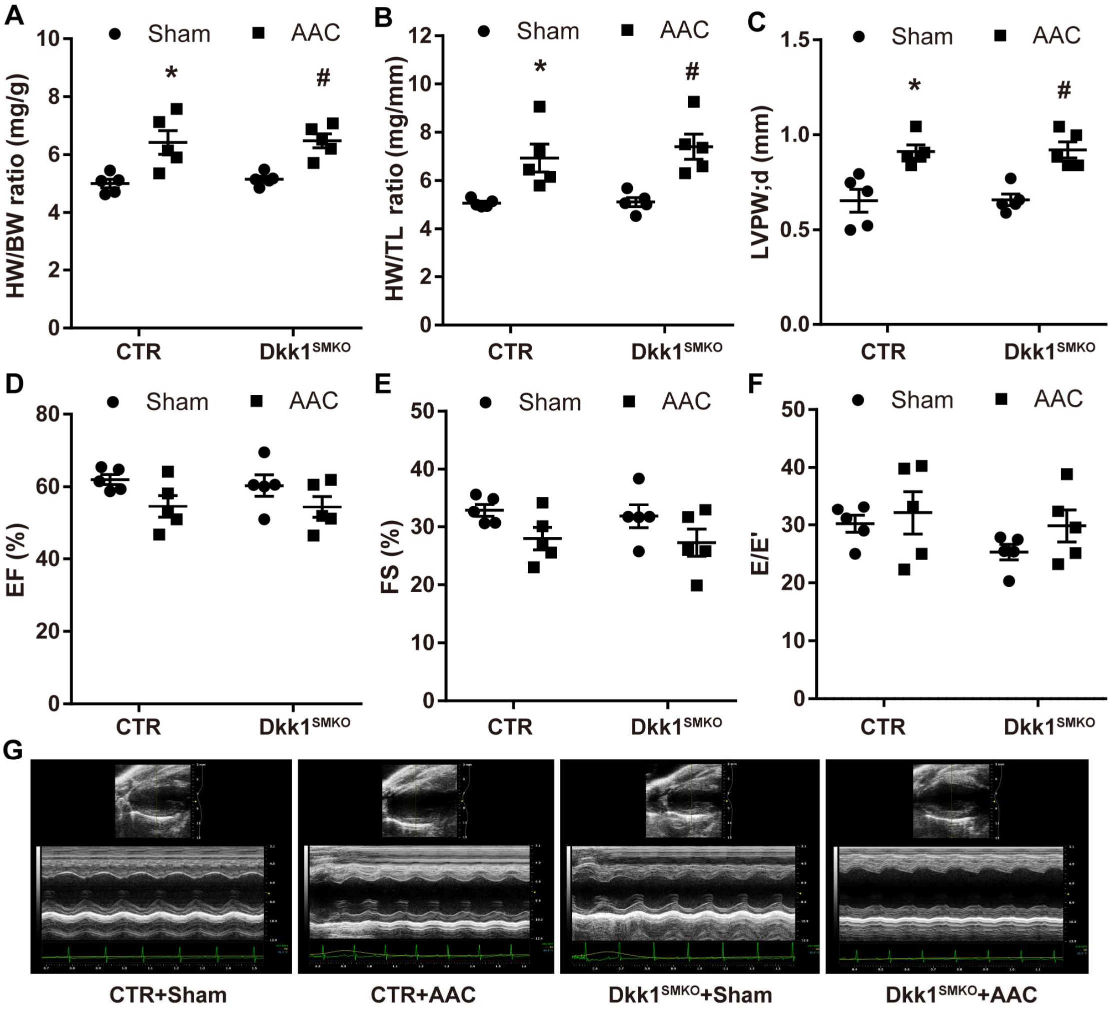

Figure S9. (**A**) Heart weight/body weight ratio. (**B**) Heart weight/tibial length ratio. Heart function was analyzed by echocardiography, and LVPW (**C**), EF% (**D**), FS% (**E**) and E/E' (**F**) were plotted. (**G**) Two-dimensional M-mode echocardiograms are shown. n= 5. *P<0.05 versus the CTR+Sham group; #P<0.05 versus the Dkk1^{SMKO}+Sham group. HW, heart weight; BW, body weight; TL, tibia length; LVPW, left ventricular posterior wall thickness; EF%, left ventricular ejection fraction; FS%, fractional shortening; E/E', the ratio of mitral peak velocity of early filling (E) to early diastolic mitral annular velocity (E').

Figure S10

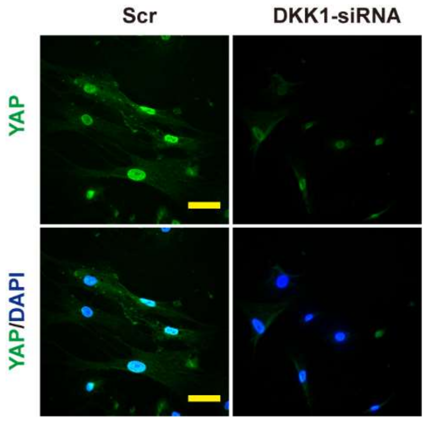

Figure S10. Knockdown or knockout of DKK1 reduces nuclear YAP localization. HASMCs were transfected with scrambled siRNA (Scr) or DKK1-siRNA for 48 hr and treated with high-level stretch (18%) for 12 hr. Then, cells were fixed and analyzed by immunofluorescence using anti-YAP antibodies (green). Nuclei were counterstained with DAPI (blue).
